# Longitudinal Association Between the Change in Serum Procollagen Type III *N*-Terminal Peptide (PIIINP) and Skeletal Muscle Loss in Patients with Gastrointestinal Cancer Receiving Chemotherapy: A Prospective Cohort Study

**DOI:** 10.3390/nu18193149

**Published:** 2026-09-24

**Authors:** Mehmet Erdinç Esenkaya, Özlem Özdemir, Mustafa Değirmenci, Kaan Kuzu, Kübra Ekiz Seyhan, Gürkan Gül

**Affiliations:** 1 Department of Medical Oncology, Izmir City Hospital, Izmir 35540, Türkiye; ozdemirozlem.md@gmail.com (Ö.Ö.); degirmencigil@gmail.com (M.D.); gurkangl6290@gmail.com (G.G.); 2 Department of Medical Biochemistry, Izmir City Hospital, Izmir 35540, Türkiye; kaan_kuzu_2042@hotmail.com (K.K.); kubraekiiz@gmail.com (K.E.S.)

**Keywords:** sarcopenia, gastrointestinal cancer, skeletal muscle index, procollagen type III *N*-terminal peptide (PIIINP), collagen turnover, bioelectrical impedance analysis, chemotherapy, muscle loss

## Abstract

**Background/Objectives:** Sarcopenia predicts increased morbidity and mortality in gastrointestinal (GI) cancer. This study evaluated the longitudinal association between changes in the skeletal muscle index (SMI) and serum procollagen type III *N*-terminal peptide (PIIINP), together with sarcopenia and malnutrition screening tools (SARC-F, SARC-CalF, MUST), during chemotherapy for GI cancer. **Methods:** In this prospective single-center study, 170 patients with GI cancer were assessed at baseline and month 6; 48 died, and longitudinal analyses included the 122 survivors. SMI was derived from bioelectrical impedance analysis (Tanita SC-330) using the Janssen equation, and serum PIIINP was measured at both time points. The primary endpoint was the association between the percentage changes in SMI and PIIINP. **Results:** BMI, SMI, and handgrip strength decreased and SARC-F and SARC-CalF scores increased significantly (all *p* < 0.001); the prevalence of low muscle mass rose from 29% to 52%, and PIIINP increased significantly. The percentage changes in SMI and PIIINP were strongly inversely correlated (ρ = −0.733, *p* < 0.001). In the parsimonious multivariable model, the percentage change in PIIINP (β = −0.47) and month-6 albumin (β = 0.31) were independently associated with the change in SMI (R^2^ = 0.37); the albumin association was model-dependent and not retained in the expanded model. **Conclusions:** In patients with GI cancer receiving chemotherapy, progressive muscle loss over six months was accompanied by a rise in the circulating collagen turnover marker PIIINP. Because the study was observational, restricted to survivors, and based on a non-muscle-specific marker, causal and muscle-specific inferences are unwarranted; PIIINP nonetheless merits further evaluation as a monitoring biomarker.

## 1. Introduction

Sarcopenia is a progressive skeletal muscle disorder characterized by reduced skeletal muscle mass, muscle strength, and physical performance; although current consensus definitions emphasize muscle strength as the primary diagnostic criterion, low muscle mass remains a central parameter for confirming the diagnosis and for serial monitoring of the condition [1,2]. Although initially described as an age-related condition, it is now recognized to develop secondary to inflammatory systemic diseases, particularly malignancy [1]. Gastrointestinal (GI) cancers (esophageal, gastric, colorectal, small bowel, biliary tract, and pancreatic tumors) are characterized by catabolic muscle loss driven by tumor-derived cytokines and systemic inflammation; sarcopenia is common in this group and deepens as the disease progresses [3]. In patients with cancer, sarcopenia is independently associated with chemotherapy toxicity, postoperative complications, and worse survival [4,5,6].

Although frequently used interchangeably, several overlapping constructs should be distinguished. Sarcopenia is a muscle disorder defined primarily by low muscle strength, with low muscle mass required for confirmation [1]. Skeletal muscle loss refers specifically to a reduction in muscle mass over time and is a component of, but not synonymous with, sarcopenia. Malnutrition denotes a state of deficient nutrient intake or assimilation leading to altered body composition and impaired function [7]. Cancer cachexia is a multifactorial syndrome characterized by ongoing loss of skeletal muscle mass, with or without loss of fat mass, driven by tumor-related inflammation and metabolic derangement and not fully reversible by conventional nutritional support [8]. These conditions coexist and overlap in patients with cancer; the present study focuses specifically on the longitudinal loss of skeletal muscle mass and its relationship with circulating procollagen type III *N*-terminal peptide (PIIINP).

Sarcopenia involves not only abnormalities in muscle quantity but also alterations in muscle quality and function [1]. Skeletal muscle fibrosis is characterized by excessive accumulation of extracellular matrix (ECM), particularly collagen, in which transforming growth factor-β (TGF-β)/Smad3 signaling plays a central role; this fibrotic remodeling can impair muscle quality and function independently of muscle quantity, so that a decline in muscle performance may not be fully captured by measures of muscle mass alone [9]. In cancer cachexia, fibrotic remodeling of skeletal muscle may be further promoted by macrophages and fibro-adipogenic progenitor (FAP) cells [10], while TGF-β activation and increased collagen deposition have also been demonstrated in experimental models of cancer-associated cachexia [11]. However, because a substantial proportion of this evidence derives from preclinical models and cachexia populations, its extrapolation to longitudinal skeletal muscle loss in broader cancer populations should be interpreted with caution [10,11]. Collectively, these observations indicate that cancer-associated muscle loss may not be explained by muscle atrophy alone and that ECM remodeling and fibrotic processes may accompany the reduction in muscle mass. Therefore, in addition to quantitative assessment of muscle mass, a circulating marker of type III collagen turnover such as PIIINP—released during type III collagen synthesis—may provide complementary information regarding the fibrotic remodeling that accompanies cancer-associated muscle loss [12].

Because sarcopenia is frequently present even before treatment and independently predicts adverse outcomes, its early and reliable detection is a clinical priority [4,6,13]. Muscle mass can be assessed objectively by dual-energy X-ray absorptiometry (DXA), bioelectrical impedance analysis (BIA), or computed tomography (CT) at the level of the third lumbar vertebra [1,2]; however, cost, the need for specialized equipment, limited accessibility, and, for some methods, radiation exposure make these techniques impractical for routine, repeated assessment [14]. This has motivated interest in practical, accessible tools, including simple low- or zero-cost equations for estimating appendicular skeletal muscle mass, for identifying and monitoring muscle depletion over time [15]. The reliability of BIA may also be reduced by altered hydration status and cachexia [16]. Against this background, the five-item SARC-F (strength, assistance with walking, rising from a chair, climbing stairs, and falls) questionnaire has become a widely used screening tool [17], although its diagnostic performance is limited: it correlates weakly with objective muscle-mass measurements, may be influenced by sex, and has low sensitivity [14,18,19], and its reliance on self-report restricts its use in severely ill or cognitively impaired patients [17]. SARC-CalF (SARC-F plus calf circumference) adds an objective component to improve screening performance [19], and, because sarcopenia, malnutrition, and cachexia overlap in patients with cancer [18], malnutrition screening tools such as the Malnutrition Universal Screening Tool (MUST) may complement this assessment [20]. None of these tools directly reflect qualitative or fibrotic changes in muscle tissue, and the relationship between changes in screening scores and objectively measured muscle loss over time, particularly during chemotherapy, has not been sufficiently investigated. Consequently, an easily accessible and objective circulating biomarker reflecting structural change in muscle is needed.

PIIINP is released into the circulation during type III collagen synthesis and has long been used in the noninvasive assessment of liver fibrosis as a component of the Enhanced Liver Fibrosis (ELF) test [12,21]. Because it may reflect extracellular matrix remodeling and fibrotic changes in skeletal muscle, it has attracted interest in recent years as a potential biomarker of muscle health [21]. In independent aging cohorts (the Long Life Family Study [LLFS], InCHIANTI, and the Korean Frailty and Aging Cohort Study), higher circulating PIIINP levels were consistently associated with poorer physical performance; in the longitudinally designed LLFS and InCHIANTI cohorts, they also predicted progressive deterioration in physical function and muscle density [21,22,23]. By contrast, the association of PIIINP with muscle mass and grip strength is less consistent; indeed, no significant association with handgrip strength was found in the Korean cohort [23]. In the oncological population, Gherghescu et al. showed that PIIINP levels were markedly higher in patients with concurrent pancreatic cancer and sarcopenia compared with healthy controls and suggested that, when evaluated together with CT-based muscle indices, PIIINP may contribute to the detection of sarcopenia [24]. Nevertheless, PIIINP is not a skeletal muscle-specific marker; as a general indicator of systemic type III collagen turnover, it may also reflect remodeling processes in other tissues, particularly hepatic fibrosis [12]. Therefore, in patients with advanced-stage cancer, in whom hepatic metastases and liver involvement are common, a rise in circulating PIIINP may partly reflect hepatic rather than skeletal-muscle collagen turnover [12], and PIIINP levels should therefore be interpreted with corresponding caution.

To date, much of the available evidence regarding the association of PIIINP with sarcopenia and physical function has been derived from healthy aging cohorts [21,22,23] or from a single cross-sectional study conducted in a single tumor type [24]. Prospective data examining the relationship between PIIINP levels and changes in muscle mass over time during chemotherapy are limited. In particular, studies encompassing multiple types of gastrointestinal cancer and jointly assessing clinical screening tools together with nutritional status are scarce. Monitoring changes in muscle mass during chemotherapy is clinically relevant, as reduced muscle mass and muscle loss have been associated with increased treatment-related toxicity and adverse clinical outcomes in patients with cancer [13,25]. Therefore, longitudinal assessment may provide clinically relevant information beyond a single baseline measurement. To our knowledge, this is the first prospective, longitudinal study to evaluate the association between the change in serum PIIINP and the change in objectively measured skeletal muscle mass during chemotherapy in a gastrointestinal cancer cohort encompassing multiple tumor types, while concurrently assessing sarcopenia and malnutrition screening tools and nutritional status. Accordingly, in the present study, skeletal muscle index (SMI), handgrip strength, sarcopenia screening scores (SARC-F and SARC-CalF), malnutrition risk (MUST), and serum PIIINP levels were prospectively evaluated at baseline and at 6 months in 170 patients with gastrointestinal cancer receiving chemotherapy. In addition, the changes in these parameters and the associations among them were examined. It was hypothesized that, over six months of chemotherapy, an increase in serum PIIINP would be accompanied by a decrease in SMI—that is, that the longitudinal changes in PIIINP and SMI would be inversely related. The secondary aims were to evaluate changes in SARC-F and SARC-CalF scores together with nutritional and inflammatory parameters during chemotherapy, to examine the association of SARC-F, SARC-CalF, and MUST results with BIA-defined low muscle mass, and to identify factors independently associated with the change in SMI.

## 2. Materials and Methods

### 2.1. Study Design

This was a prospective, longitudinal, single-center observational cohort study conducted at the Medical Oncology Clinic of Izmir City Hospital (Izmir, Türkiye), designed and reported in accordance with the Strengthening the Reporting of Observational Studies in Epidemiology (STROBE) statement for cohort studies [26]. Consecutive patients with histopathologically confirmed gastrointestinal cancer who were scheduled to begin a systemic chemotherapy regimen were enrolled between 1 October 2024 and 1 October 2025. No study-specific treatment was administered; patients were followed prospectively while continuing their standard oncological care.

Each participant was assessed at two prespecified time points: at enrollment, immediately before the initiation of the index chemotherapy regimen (baseline), and six months later (month 6). The six-month interval was chosen a priori as a pragmatic monitoring window—long enough to capture a measurable change in muscle mass and collagen turnover during systemic treatment, while remaining clinically feasible, and consistent with a period over which chemotherapy-associated muscle loss in gastrointestinal cancer has been shown to be detectable and prognostically relevant [25].

All patients remained under systemic chemotherapy during the six-month follow-up unless follow-up was terminated by death; no surviving patient permanently discontinued systemic treatment during this period. Consistent with its real-world design, the cohort was not protocol-standardized: patients received different regimens with adjuvant, neoadjuvant, or palliative intent, and some were enrolled at first line whereas others began a subsequent line.

The study protocol was approved by the Non-Interventional Clinical Research Ethics Committee of Izmir City Hospital (decision no. 2024/92; date of approval 21 August 2024). All procedures accorded with the Declaration of Helsinki and its amendments; written informed consent was obtained from all participants before any study-specific assessment.

### 2.2. Participants

The inclusion criteria were as follows: age 18 years or older; a histopathologically confirmed diagnosis of gastrointestinal cancer; a planned initiation of a systemic chemotherapy regimen (first-line or a subsequent line); and the availability of a complete baseline laboratory workup performed as part of routine pre-chemotherapy care, comprising a complete blood count, liver and renal function tests, and serum electrolytes, together with serum *C*-reactive protein (CRP) and albumin measurements and a baseline serum sample obtained for PIIINP measurement, all collected before initiation of the index chemotherapy regimen. The exclusion criteria were as follows: pregnancy or breastfeeding; a concurrent second primary malignancy outside the gastrointestinal tract; neurodegenerative disease (including Alzheimer’s disease); muscular dystrophy; end-stage chronic kidney disease or cirrhosis; and any acute condition capable of eliciting a systemic acute-phase inflammatory response within the four weeks preceding assessment, including a clinically significant infection (defined as an active infection requiring systemic antimicrobial therapy or hospitalization, such as pneumonia, sepsis, febrile neutropenia, or symptomatic urinary tract infection), major trauma or surgery, or another acute inflammatory event (e.g., acute pancreatitis, venous thromboembolism, acute coronary syndrome, or an acute flare of a chronic inflammatory or autoimmune disease). These conditions were excluded to minimize the potential influence of acute systemic inflammation on the study measurements. Patients with non-end-stage chronic kidney disease were eligible for inclusion. Patients who declined to participate in the study were not evaluated.

During the study period (1 October 2024–1 October 2025), 170 patients who met the eligibility criteria were enrolled, and all were assessed before the initiation of the index chemotherapy regimen. Enrollment was based on consecutive eligible patients who provided written informed consent; the exact numbers of individuals screened for eligibility and of those who declined participation were not systematically recorded. Over the six-month follow-up period, 48 patients (28%) died. No loss to follow-up occurred other than death; all 122 surviving patients completed both the baseline and month-6 assessments. Therefore, the longitudinal analyses were conducted on these 122 patients.

### 2.3. Data Collection

All data were recorded on a standardized case report form through clinical assessment and patient records. Comorbidities were ascertained from physician-documented diagnoses in the medical records rather than by de novo protocolized screening, which may underestimate the true prevalence of undiagnosed conditions such as hypertension. At baseline, the following were recorded: demographic characteristics (age and sex), comorbidities (hypertension, diabetes mellitus, coronary artery disease, chronic obstructive pulmonary disease, and chronic kidney disease), primary tumor location, disease stage determined according to the American Joint Committee on Cancer (AJCC) Cancer Staging System (8th Edition) [27], the intent of chemotherapy (adjuvant, neoadjuvant, or palliative), and the administered chemotherapy regimen. Anthropometric measurements (body weight and height, and the body mass index [BMI] derived from them) were obtained at baseline and at month 6. BMI was categorized as underweight (<18.5 kg/m^2^), normal weight (18.5–24.9 kg/m^2^), overweight (25.0–29.9 kg/m^2^), or obese (≥30.0 kg/m^2^) according to World Health Organization criteria [28]. As part of routine pre-chemotherapy care, all patients underwent a complete baseline laboratory workup, including a complete blood count, liver and renal function tests, and serum electrolytes. Of these routine laboratory parameters, serum creatinine, *C*-reactive protein (CRP), and albumin, together with the standard liver function tests (alanine aminotransferase [ALT], aspartate aminotransferase [AST], alkaline phosphatase [ALP], gamma-glutamyl transferase [GGT], and total bilirubin), were included in the present analyses and were measured in all patients at both baseline and month 6 using identical methods. Serum creatinine, CRP, and albumin concentrations were measured using a Roche cobas c 701/702 analyzer (Roche Diagnostics, Mannheim, Germany). Creatinine was determined using the Creatinine Jaffé Gen.2 assay by a kinetic colorimetric Jaffé method; CRP was measured using the Tina-quant *C*-Reactive Protein IV assay by a particle-enhanced immunoturbidimetric method; and albumin was determined using the Albumin Gen.2 assay by a bromocresol green (BCG)-based colorimetric method. The liver function tests were measured at both time points on the same Roche cobas c 701/702 platform using the manufacturer’s standardized enzymatic and colorimetric assays. The presence of liver metastasis at baseline was recorded as present or absent from routine staging imaging (computed tomography, magnetic resonance imaging, and/or positron-emission tomography) as documented in the corresponding radiology reports. Serum PIIINP was separately measured at both time points as part of the study protocol. Sarcopenia parameters were likewise assessed at both time points using the methods described in the following sections. Corticosteroids were administered as part of standard chemotherapy premedication and supportive care when indicated; however, cumulative corticosteroid dose and duration of exposure were not systematically recorded. Chemotherapy-related toxicity and disease progression during the six-month follow-up were not prospectively collected as standardized study variables. All month-6 study assessments—anthropometry, bioelectrical impedance analysis, and blood sampling for PIIINP and routine laboratory parameters—were performed at a single visit on the day before a scheduled chemotherapy infusion.

### 2.4. Assessment of Sarcopenia

#### 2.4.1. Bioelectrical Impedance Analysis (BIA)

Skeletal muscle mass was assessed by bioelectrical impedance analysis (BIA). Measurements were performed at baseline and at month 6 using a body composition analyzer (Tanita SC-330, Tanita Corp., Tokyo, Japan), and the electrical resistance (R) value was recorded in ohms. All BIA measurements were performed under standardized conditions in accordance with established recommendations [16,29]. Measurements were obtained using the same device (Tanita SC-330) by a single trained operator at both the baseline and month-6 visits, in the morning, after the patient had emptied their bladder, and at least 2 h after the last meal or fluid intake. Patients were instructed to avoid strenuous physical activity, alcohol, and caffeine in the hours preceding the assessment. Measurements were performed with patients lightly dressed, without metallic objects, at room temperature, and positioned according to the manufacturer’s instructions. For each patient, baseline and month-6 measurements were obtained under the same conditions and at approximately the same time of day to minimize within-subject variability. These standardized pre-measurement conditions were applied to minimize the potential influence of short-term variations in hydration status on BIA-derived estimates. Because month-6 assessments were scheduled on the day before an infusion (Section 2.3), the potential effect of acute intravenous hydration on bioimpedance measurements was minimized.

The Janssen equation was chosen because it was developed and cross-validated against magnetic resonance imaging-measured skeletal muscle mass in a large, heterogeneous adult population and provides a practical, radiation-free, low-cost, and readily repeatable bioimpedance-based estimate of whole-body skeletal muscle mass [30]. This published equation was used rather than the analyzer’s proprietary body-composition output, which relies on undisclosed, manufacturer-specific algorithms, in order to ensure methodological transparency and reproducibility and to enable comparison with other studies reporting Janssen-derived SMI. Skeletal muscle mass (SMM) was calculated using the equation developed and validated by Janssen et al. [30]:

SMM (kg) = [0.401 × (height^2^/R) + 3.825 × sex − 0.071 × age] + 5.102

In this equation, height was entered in centimeters, R in ohms, and age in years; sex was coded as 1 for men and 0 for women. The skeletal muscle index (SMI) was calculated by dividing the SMM value by the square of height in meters:

SMI (kg/m^2^) = SMM/height (m)^2^

For BIA-based classification of low muscle mass, SMI values of <10.76 kg/m^2^ in men and <6.76 kg/m^2^ in women were used as the sex-specific thresholds [31].

Two distinct constructs were assessed. Cross-sectional low muscle mass at each time point was defined using the sex-specific Janssen SMI cutoffs stated above. Skeletal muscle loss over time was defined as a longitudinal decline in SMI between baseline and month 6 and was quantified both as the absolute change (ΔSMI, kg/m^2^) and the percentage change (%ΔSMI = [(month-6 SMI − baseline SMI)/baseline SMI] × 100), with negative values indicating muscle loss. Muscle loss was analyzed primarily as a continuous variable rather than dichotomized, and the percentage change in SMI constituted the primary endpoint.

#### 2.4.2. Handgrip Strength

Muscle strength was assessed at baseline and month 6 using a handgrip dynamometer (TAKEI 5401, Takei Scientific Instruments Co., Ltd., Niigata, Japan). Measurements were performed using a standardized protocol with the patient seated, the shoulder adducted and in a neutral position, the elbow flexed at 90°, the forearm in a neutral position, and the wrist in a comfortable neutral position. Handgrip strength was assessed using the dominant hand. After a brief familiarization with the procedure, patients were instructed to squeeze the dynamometer as strongly as possible. Three maximal attempts were performed, with a short rest interval between attempts, and the highest value was recorded in kilograms. The same protocol was applied at baseline and month 6. Handgrip strength was analyzed as a continuous variable (kg), without applying a categorical threshold.

#### 2.4.3. Screening Tools

Malnutrition risk was assessed using the Malnutrition Universal Screening Tool (MUST). MUST consists of the sum of subscores relating to body mass index, unintentional weight loss, and the effect of acute illness. Based on the total score, patients were categorized as being at low (score 0), medium (score 1), or high (score ≥ 2) risk of malnutrition [20].

Sarcopenia risk was assessed using the SARC-F and SARC-CalF screening tools. SARC-F evaluates five components—muscle strength, walking, rising from a chair, climbing stairs, and history of falls; each component is scored from 0 to 2, and the total score ranges from 0 to 10. A total score of ≥4 was considered a positive screen (high sarcopenia risk) [32]. SARC-CalF is a modified version created by adding calf circumference measurement to SARC-F. Calf circumference was measured in centimeters at the widest point of the calf using a non-stretchable tape measure, with the patient standing and weight distributed equally on both legs. In accordance with the original SARC-CalF scoring, 10 points were added to the score when calf circumference was ≤34 cm in men and ≤33 cm in women; thus, the total score was evaluated over a range of 0–20, and a score of ≥11 was considered a positive screen (high sarcopenia risk) [19]. Validated Turkish versions of both scales, established through validity and reliability studies, were used [33].

A single composite operational definition of sarcopenia was not applied (e.g., the algorithm of the revised European Working Group on Sarcopenia in Older People [EWGSOP2], in which low muscle strength indicates probable sarcopenia and low muscle quantity or quality is used to confirm the diagnosis) [1]. Instead, low muscle mass was defined using BIA-derived SMI according to the sex-specific cutoffs described above, while muscle strength was assessed separately by handgrip dynamometry as a continuous variable. Sarcopenia risk was screened using SARC-F and SARC-CalF. Accordingly, the BIA-based classification represents low muscle mass rather than a confirmed diagnosis of sarcopenia.

#### 2.4.4. Assessment of Systemic Inflammation

Systemic inflammation was assessed using the modified Glasgow Prognostic Score (mGPS). The mGPS was determined at both baseline and month 6 using serum *C*-reactive protein (CRP) and albumin concentrations obtained at the corresponding time point. The mGPS was calculated based on serum CRP and albumin levels: patients with CRP ≤ 10 mg/L were assigned 0 points regardless of albumin level; patients with CRP > 10 mg/L and albumin ≥ 35 g/L were assigned 1 point; and patients with CRP > 10 mg/L and albumin < 35 g/L were assigned 2 points [34].

### 2.5. Measurement of Serum PIIINP Levels

Serum PIIINP levels were measured using a commercially available sandwich enzyme-linked immunosorbent assay (ELISA) kit (Elabscience^®^ (Wuhan, China) Human PIIINP ELISA Kit, Cat. No. E-EL-H0183) according to the manufacturer’s instructions. All assays were performed by laboratory personnel who were blinded to the participants’ bioimpedance and clinical data. The analytical sensitivity of the assay was 14.06 pg/mL, with a measurement range of 23.44–1500 pg/mL. The reported intra-assay and inter-assay coefficients of variation were 3.61–6.37% and 3.59–5.59%, respectively, both below 10%. Absorbance was read at 450 nm using a PR 4100 microplate reader (Bio-Rad Laboratories, Hercules, CA, USA). Serum samples were stored at −80 °C until analysis, with a maximum storage duration of approximately 3 months. Grossly hemolyzed samples were not analyzed and were replaced by newly collected serum samples from the corresponding patients; the exact number of re-collected samples was not recorded. All samples were analyzed in duplicate, and the mean of the two measurements was used for statistical analyses. Samples with concentrations exceeding the upper limit of the standard curve were diluted 1:20 using the Reference Standard & Sample Diluent supplied with the kit and reanalyzed. Final PIIINP concentrations were calculated after accounting for the corresponding dilution factor. Baseline and month-6 samples from the same patient were analyzed in separate ELISA runs; the low inter-assay coefficient of variation (<5.6%) limits the potential impact of between-run variability on the measured longitudinal change. Because PIIINP is not internationally standardized, absolute concentrations obtained with this research-use ELISA are assay-specific and are not directly comparable with values reported by clinical Enhanced Liver Fibrosis (ELF) immunoassay platforms; accordingly, the analyses focused on within-patient longitudinal changes rather than on absolute concentrations.

### 2.6. Study Endpoints

The primary endpoint of the study was to evaluate the association between the percentage change in the skeletal muscle index (SMI) and the percentage change in serum PIIINP levels between baseline and month 6. For each parameter, the percentage change was calculated as follows:

Percentage change = [(month-6 value − baseline value)/baseline value] × 100

The secondary endpoints were as follows: (i) evaluation of changes in anthropometric, functional, nutritional, and laboratory parameters (BMI, handgrip strength, SARC-F, SARC-CalF, MUST, mGPS, creatinine, CRP, and albumin) between baseline and month 6; (ii) evaluation of the association between the malnutrition and sarcopenia screening tools (MUST, SARC-F, and SARC-CalF) and BIA-based low muscle mass at baseline and at month 6; (iii) evaluation of the association between the percentage change in SMI and clinical, biochemical, and screening parameters; and (iv) identification of factors associated with the percentage change in SMI by multivariable linear regression analysis.

### 2.7. Statistical Analysis

Statistical analyses were performed using SPSS software (Version 26.0, IBM Corp., Armonk, NY, USA), jamovi (Version 2.7; The jamovi Project, 2025, using the R statistical computing environment, Version 4.5, and the R packages *car* and *moretests* for regression diagnostics), and MedCalc (Version 23.0.2; MedCalc Software Ltd., Ostend, Belgium). A two-sided *p* value < 0.05 was considered statistically significant in all analyses. Categorical variables were presented as frequencies and percentages; continuous variables were presented, according to their distribution, as mean ± standard deviation or median (interquartile range, IQR). The normality of the distribution of continuous variables was assessed using the Shapiro–Wilk test together with visual methods (histogram and Q–Q plot).

Baseline data were complete for all 170 enrolled patients, and no loss to follow-up occurred other than death. Longitudinal analyses were performed on the 122 patients who completed both assessments using an available-case approach, without imputation; month-6 values were missing only for body mass index in 2 patients and for handgrip strength in 3 patients, so that the corresponding paired analyses were based on 120 and 119 patients, respectively.

For the comparison of baseline and month-6 measurements, the paired-samples *t*-test was used for normally distributed variables and the Wilcoxon signed-rank test for non-normally distributed variables, with the Hodges–Lehmann estimator and its 95% confidence interval reported alongside the Wilcoxon test as the corresponding measure of absolute change; the change in the mGPS and MUST risk-category distributions was assessed with the McNemar–Bowker test, and the change in SARC-F and SARC-CalF risk categories with the McNemar test. The association between the screening tools (MUST, SARC-F, SARC-CalF) and SMI categories was examined using the Pearson chi-square test when expected frequencies were adequate, the chi-square test with Yates continuity correction for 2 × 2 tables, the Fisher exact test when the expected cell frequency was below 5, and the Fisher–Freeman–Halton exact test for larger contingency tables in which more than 20% of cells had an expected count below 5.

To assess potential survivor bias, baseline characteristics were compared between patients who survived to month 6 (*n* = 122) and those who died during the six-month follow-up (*n* = 48), using the independent-samples *t*-test or the Mann–Whitney U test for continuous variables and the Pearson chi-square test, chi-square test with Yates’ continuity correction, Fisher’s exact test, or Fisher–Freeman–Halton exact test for categorical variables, as appropriate to the expected cell counts (Supplementary Tables S1 and S2).

The associations between the percentage change in SMI and other parameters were assessed using Spearman correlation analysis; the correlation coefficient (rho) was interpreted as very weak for 0.00–0.19, weak for 0.20–0.39, moderate for 0.40–0.59, strong for 0.60–0.79, and very strong for 0.80–1.00, with negative values indicating an inverse association. To account for multiple comparisons, the resulting *p* values were adjusted using the Benjamini–Hochberg false discovery rate (FDR) procedure [35]. As a sensitivity analysis to assess the potential influence of chronic kidney disease on the primary association, the correlation between the percentage changes in SMI and PIIINP was repeated after excluding patients with chronic kidney disease. To evaluate a potential hepatic contribution to the observed PIIINP–SMI association, three additional analyses were performed. First, the Spearman correlation between the percentage changes in SMI and PIIINP was repeated separately in patients with and without baseline liver metastasis. Second, the Spearman correlation between the percentage change in PIIINP and the six-month change in each liver function test (ALT, AST, ALP, GGT, and total bilirubin) was examined. Third, baseline and month-6 liver function tests were compared using the Wilcoxon signed-rank test to determine whether hepatic function changed appreciably over the follow-up period.

Multiple linear regression analysis was performed to identify variables independently associated with the percentage change in SMI. Two models were fitted using the enter method: Model 1 (parsimonious), including the percentage change in PIIINP, month-6 albumin, and month-6 CRP, corresponding to the originally reported model, and Model 2 (expanded), additionally adjusting for age, sex, disease stage, baseline SMI, baseline PIIINP, treatment intent, and primary tumor site, and substituting baseline albumin and baseline CRP for the month-6 values to reduce the possibility of reverse-causation bias. Because Model 2 substitutes predictors rather than simply adding covariates to Model 1, the two models are not nested and were therefore evaluated independently rather than compared with a formal F-change test. Model assumptions (linearity, normality, and homoscedasticity) were checked with residual analyses (Shapiro–Wilk, Breusch–Pagan, Goldfeld–Quandt, Harrison–McCabe, and Durbin–Watson tests); multicollinearity was assessed using the variance inflation factor (VIF), with values < 5 interpreted as indicating no relevant multicollinearity. A sensitivity analysis was performed using natural log-transformed PIIINP and SMI values (Δln[PIIINP], Δln[SMI]), with the same covariates as Model 1, to address the right-skewed distribution of the percentage-change variables (Supplementary Table S3). The explanatory power of the models was evaluated with R^2^ and adjusted R^2^, and their significance with the F test. To directly assess a potential hepatic contribution to the observed association, the expanded model (Model 2) was additionally re-fitted with each baseline liver function test (ALT, AST, ALP, GGT, and total bilirubin) entered in turn as an additional covariate. Each enzyme was entered separately rather than simultaneously to avoid increasing the number of model parameters and to preclude collinearity between functionally related enzymes (ALT–AST and ALP–GGT). To assess whether the PIIINP coefficient in the expanded model was robust to the number of parameters relative to the sample size, two penalized regression models were additionally fitted to the Model 2 predictor set—ridge regression and least absolute shrinkage and selection operator (LASSO) regression—each with the shrinkage parameter selected by 10-fold cross-validation.

## 3. Results

### 3.1. Study Population

A total of 170 patients with gastrointestinal cancer were enrolled in the study (Figure 1). Over the six-month follow-up period, 48 patients (28%) died. Of these deaths, 11 (22.9%) occurred within the first three months of follow-up and 37 (77.1%) occurred between months 3 and 6. Cause-specific mortality data were not systematically collected as part of the study protocol and therefore could not be reliably reported. Consequently, the longitudinal analyses were performed on 122 patients who completed both the baseline and month-6 assessments.

To evaluate potential survivor bias, baseline characteristics were compared between the 122 survivors and the 48 patients who died during follow-up (Tables S1 and S2). Patients who died were significantly older and had a less favorable baseline inflammatory, nutritional, and functional-screening profile (higher CRP, lower albumin, a less favorable mGPS distribution, and higher SARC-F and SARC-CalF scores; all *p* ≤ 0.005). In contrast, baseline skeletal muscle mass did not differ between the groups—whether expressed as SMI (10.13 vs. 10.14 kg/m^2^, *p* = 0.980) or as the prevalence of low muscle mass (*p* = 0.886)—and baseline serum PIIINP, handgrip strength, disease stage, tumor site, treatment intent, and chemotherapy regimen were likewise comparable (all *p* > 0.05).

### 3.2. Baseline Characteristics

The demographic, clinical, and oncological characteristics of the 122 patients included in the longitudinal analysis are summarized in Table 1 and Table 2. The cohort had a mean age of 60.3 ± 10.4 years and was predominantly male (67%), with a mean baseline body mass index of 24.8 ± 4.8 kg/m^2^. Colorectal tumors predominated (colon, 48%; rectal, 21%), followed by gastric cancer (21%); most patients had stage IV disease (64%) and were treated with palliative intent (65%), most frequently with FOLFOX (folinic acid, fluorouracil, and oxaliplatin; 56%).

### 3.3. Changes Observed During Six-Month Follow-Up

Over the six-month follow-up, the skeletal muscle index declined significantly (from 10.14 ± 1.75 to 9.50 ± 1.76 kg/m^2^; mean change −0.64 ± 0.80 kg/m^2^, −6.2% ± 7.1%; *p* < 0.001), whereas serum PIIINP increased (from 1522 ± 901 to 2072 ± 889 pg/mL; +550 ± 552 pg/mL, +71% ± 96%; *p* < 0.001). In parallel, the prevalence of BIA-defined low muscle mass increased from 35 patients (29%) at baseline to 64 (52%) at month 6. Body mass index and handgrip strength also decreased significantly, and both SARC-F and SARC-CalF scores increased (all *p* < 0.001). Among laboratory parameters, *C*-reactive protein increased and albumin decreased, and the mGPS distribution shifted toward higher scores, whereas creatinine remained unchanged (Table 3 and Table 4).

### 3.4. Screening Tools and SMI Category

The associations between the malnutrition and sarcopenia screening categories and the low muscle mass status defined by SMI are shown in Table 5. At baseline, none of the screening tools were significantly associated with SMI category (MUST, *p* = 0.798; SARC-F, *p* = 0.103; SARC-CalF, *p* = 0.827). This lack of association persisted at month 6, when none of the tools were significantly associated with SMI category (MUST, *p* = 0.465; SARC-F, *p* = 0.491; SARC-CalF, *p* = 0.479).

### 3.5. Correlation Analyses

Factors associated with the percentage change in SMI were assessed by Spearman correlation (Table 6). The strongest association was a strong inverse correlation between the percentage changes in SMI and PIIINP (ρ = −0.733, *p* < 0.001; Figure 2, which also shows the corresponding Pearson coefficient, r = −0.55). The correlation between the absolute changes in SMI and PIIINP was of similar magnitude (ρ = −0.722, *p* < 0.001; Table 6), indicating that the association was not an artifact of the percentage transformation. This association remained essentially unchanged after excluding the six patients with chronic kidney disease (ρ = −0.725, *p* < 0.001). Month-6 albumin correlated positively and month-6 CRP negatively with the change in SMI (ρ = 0.479 and −0.391, respectively; both *p* < 0.001), and weaker but significant correlations were observed with the month-6 SARC-CalF, SARC-F, and handgrip values and with baseline and month-6 PIIINP (Table 6). All significant correlations retained significance after Benjamini–Hochberg FDR correction, whereas no association was found with age, BMI, creatinine, or the baseline albumin, CRP, handgrip, and screening measures (all *p* > 0.05).

### 3.6. Multivariable Linear Regression

Two multiple linear regression models were fitted to identify factors independently associated with the percentage change in SMI (Table 7). Model 1 (parsimonious), including the percentage change in PIIINP, month-6 albumin, and month-6 CRP, was significant overall (F(3, 118) = 22.75, *p* < 0.001; R^2^ = 0.37, adjusted R^2^ = 0.35); the percentage change in PIIINP (β = −0.47, *p* < 0.001) and month-6 albumin (β = 0.31, *p* = 0.001) were independently associated with the change in SMI, whereas month-6 CRP was not (β = 0.08, *p* = 0.349). Model 2 (expanded), which additionally adjusted for age, sex, disease stage, baseline SMI, baseline PIIINP, treatment intent, and primary tumor site and used baseline instead of month-6 albumin and CRP, was also significant (F(14, 107) = 6.18, *p* < 0.001; R^2^ = 0.45, adjusted R^2^ = 0.37). In Model 2, the percentage change in PIIINP (β = −0.57, *p* < 0.001) and baseline SMI (β = −0.28, *p* = 0.006) were independently associated with the change in SMI; notably, whereas month-6 albumin was an independent correlate in Model 1, baseline albumin was not significant in Model 2 (β = −0.10, *p* = 0.258), indicating that the albumin association was model-dependent. Model assumptions were satisfied and all VIF values were below 5 in both models (Figure 3). In a sensitivity analysis using natural log-transformed PIIINP and SMI values, the association was of similar magnitude (β = −0.59, *p* < 0.001; R^2^ = 0.46; Supplementary Table S3). In penalized regression analyses using the Model 2 predictor set, the standardized PIIINP coefficient remained stable: ridge regression yielded β = −0.46, and LASSO retained PIIINP as the dominant predictor (β = −0.44), together with baseline SMI and palliative treatment intent. The PIIINP coefficient was therefore consistent across the parsimonious (Model 1, β = −0.47), expanded (Model 2, β = −0.57), and penalized specifications, indicating that it was not an artifact of overfitting (Supplementary Table S4).

### 3.7. Hepatic Sensitivity Analyses

Because PIIINP is a marker of type III collagen turnover that may also be released during hepatic fibrogenesis, several analyses were performed to examine whether the observed inverse association between the six-month percentage changes in PIIINP and SMI could be attributed to hepatic factors. At baseline, 36 of the 122 patients (29.5%) had radiologically documented liver metastasis. Baseline liver function tests were within the reference range in a large majority of patients (ALT, 118/122 [96.7%]; AST, 115/122 [94.3%]; GGT, 114/122 [93.4%]; ALP, 114/122 [93.4%]; total bilirubin, 122/122 [100%]), with median values of 24.0 U/L (IQR, 18.0–29.0) for ALT, 23.0 U/L (18.0–30.0) for AST, 46.0 U/L (42.0–52.0) for GGT, 81.0 U/L (73.2–88.0) for ALP, and 0.4 mg/dL (0.3–0.5) for total bilirubin.

When the primary correlation was stratified by baseline liver-metastasis status, the inverse association between the percentage changes in PIIINP and SMI remained statistically significant in both subgroups and was numerically stronger in patients without liver metastasis (ρ = −0.812, *p* < 0.001, *n* = 86) than in those with liver metastasis (ρ = −0.485, *p* = 0.003, *n* = 36).

The percentage change in PIIINP was not correlated with the six-month change in any liver function test (ALT, ρ = −0.070, *p* = 0.440; AST, ρ = −0.008, *p* = 0.928; GGT, ρ = −0.125, *p* = 0.169; ALP, ρ = −0.137, *p* = 0.131; total bilirubin, ρ = −0.073, *p* = 0.427). Over the six-month follow-up, ALT and AST showed small increases that reached nominal significance (median, 24.0 to 24.0 U/L, *p* = 0.049; and 23.0 to 25.0 U/L, *p* = 0.014, respectively), whereas GGT, ALP, and total bilirubin did not change significantly (all *p* > 0.30); neither the ALT nor the AST change remained significant after Bonferroni correction for the five comparisons (α = 0.010), and all median values remained within the reference range throughout follow-up.

Finally, when the expanded multivariable model (Model 2) was re-fitted with each baseline liver function test entered in turn as an additional covariate, the percentage change in PIIINP remained an independent correlate of the percentage change in SMI in every model (standardized β ranging from −0.566 to −0.583; all *p* < 0.001), whereas none of the liver function tests were independently associated with the change in SMI (ALT, β = −0.077, *p* = 0.310; AST, β = −0.139, *p* = 0.073; GGT, β = −0.033, *p* = 0.667; ALP, β = −0.026, *p* = 0.725; total bilirubin, β = +0.048, *p* = 0.537). These analyses are summarized in Table 8.

## 4. Discussion

In this prospective study, the association between the change in the skeletal muscle index (SMI) and the change in levels of the collagen turnover marker procollagen type III *N*-terminal peptide (PIIINP) was evaluated longitudinally over a six-month follow-up period in patients with gastrointestinal cancer receiving chemotherapy. The main finding was a strong and inverse association between the percentage change in PIIINP levels and the percentage change in SMI. Furthermore, the percentage change in PIIINP remained the strongest independent correlate of the change in SMI across parsimonious, expanded, penalized, and log-transformed models, supporting its robustness to adjustment for measured clinical and biochemical factors and to the skewed distribution of the change variables. This finding suggests that changes in collagen turnover and extracellular matrix remodeling may accompany cancer-associated muscle loss. Moreover, because the study is observational and PIIINP and SMI were assessed concomitantly, the association between their changes should be interpreted as a temporal co-occurrence rather than as evidence that extracellular matrix remodeling causes muscle loss, and the direction of any underlying relationship cannot be determined. The independent association of baseline SMI with the subsequent change in the expanded model may partly reflect regression to the mean.

The muscle loss observed in this cohort should be interpreted within the overlap between sarcopenia and cancer cachexia, which frequently coexist in patients with gastrointestinal cancer in whom tumor burden, systemic inflammation, and reduced intake commonly converge. Muscle loss was quantified using the skeletal muscle index rather than by applying formal cachexia criteria; patients were therefore not classified as cachectic, and the observed decline in muscle mass likely reflects the combined contribution of these overlapping processes. The association between rising PIIINP and progressive muscle loss is biologically plausible across this spectrum, as increased collagen turnover and extracellular matrix remodeling are common to muscle wasting irrespective of its precise aetiological classification.

In skeletal muscle, the ECM is a fundamental component that holds muscle fibers together, mediates force transmission, and plays a critical role in repair after injury; however, fibrosis, defined as the excessive accumulation of matrix components—particularly collagen—impairs muscle function, adversely affects regeneration, and is regarded as an important cause of muscle weakness [36]. One of the principal mechanisms that may contribute to extracellular matrix remodeling and fibrosis in cancer cachexia is the TGF-β signaling pathway. Increased TGF-β expression, accompanied by dense collagen deposition and fibroblast–myofibroblast transition, has been demonstrated in the tumor tissue of cachectic patients with colorectal cancer [11]. This fibrotic process is not confined to the tumor; in cancer cachexia, the skeletal muscle extracellular matrix has also been reported to undergo remodeling and fibrosis through fibro-adipogenic progenitors and profibrotic immune cells [10]. The dual effect of TGF-β, both triggering fibrotic remodeling and contributing to muscle atrophy, suggests that fibrosis and muscle loss may share a common biological basis. In the present study, the concomitance of an increase in PIIINP levels with the decrease in SMI is consistent with this conceptual framework, which proposes a possible biological link between collagen remodeling and cancer-associated muscle loss.

An important nuance is that PIIINP may reflect not only pathological fibrosis but also anabolic muscle remodeling: administration of growth hormone and testosterone [37] and resistance exercise [38] have both been reported to raise PIIINP in parallel with gains in muscle mass. In isolation, therefore, a rise in PIIINP cannot distinguish maladaptive fibrotic from adaptive regenerative remodeling, and its interpretation depends on the clinical context. In the present cohort, patients were in a catabolic state imposed by chemotherapy and advanced disease rather than receiving an anabolic stimulus, and the increase in PIIINP was accompanied by a decrease—rather than a gain—in muscle mass, supporting an interpretation of maladaptive fibrotic remodeling. Nevertheless, because circulating PIIINP cannot distinguish whether remodeling is physiological or pathological, this remains a context-based inference rather than mechanistic evidence.

A further consideration is the specificity of PIIINP for skeletal muscle. More broadly, as a marker of type III collagen synthesis irrespective of its anatomical origin, PIIINP may also be influenced by fibrotic remodeling in other organs and by systemic factors such as age, further limiting its tissue specificity. Consequently, the observed longitudinal increase in PIIINP cannot be attributed specifically to skeletal muscle, and competing sources of collagen turnover may have contributed to the circulating PIIINP signal. Therefore, although the longitudinal association with SMI supports further investigation of PIIINP as a potential monitoring biomarker, the present findings do not establish sufficient specificity for PIIINP to be considered a skeletal muscle-specific biomarker of muscle loss.

The systemic inflammatory response plays a central role in the pathogenesis of cancer-associated muscle loss; this response, which is associated with loss of lean tissue, anorexia, fatigue, and shortened survival, is one of the defining components of cancer cachexia, and inflammation-based indices such as the modified Glasgow Prognostic Score (mGPS) have emerged as validated prognostic tools in these patients [39]. In the present study, the shift of the mGPS distribution toward higher scores during follow-up suggests that the systemic inflammatory burden increased over time in this cohort. Although *C*-reactive protein was associated with the change in muscle mass, it did not make a significant independent contribution in the multivariable model; this suggests that the effect of inflammation was largely reflected through variance shared with the nutritional and collagen turnover indicators. Albumin level, an indicator of nutritional status, declined significantly during follow-up and was independently associated with the change in muscle mass in the parsimonious model (Model 1, which used month-6 albumin); however, baseline albumin was not significant in the expanded model (Model 2; β = −0.10, *p* = 0.258), so this association should be regarded as model-dependent; this finding is consistent with the literature reporting that higher albumin levels are associated with better survival in various cancer types, particularly gastrointestinal tumors [40]. However, this association should be interpreted with caution: albumin is an indicator not only of nutritional status but also of systemic inflammation as a negative acute-phase reactant; therefore, the muscle-preserving effect of albumin cannot be attributed to nutrition alone, and at least part of it may be explained by a lower inflammatory and disease burden. Consistent with this, the causal nature of the albumin–survival association remains unproven [40]. The finding that a substantial proportion of patients were at risk of malnutrition supports the need for regular assessment and close monitoring of nutritional status in this population. However, because a controlled nutritional intervention was not applied in the present study, the efficacy or causal contribution of nutritional support in preventing muscle loss cannot be evaluated with this study design.

In clinical settings where objective measurement of muscle mass is not always practical, self-report-based functional screening tools such as SARC-F and SARC-CalF are widely used. The finding that these functional tools are associated with mortality in large cohorts supports their prognostic value [14]. In the present study, SARC-F and SARC-CalF scores worsened significantly during follow-up, indicating that these tools can reflect progressive functional deterioration over time at the group level. By contrast, the cross-sectional agreement of these same tools with the BIA-defined low muscle mass category was weak and did not reach statistical significance at either baseline or month 6. This discordance suggests that functional screening and BIA-based mass measurement assess different constructs: the former captures self-reported strength and physical performance, whereas BIA-derived SMI estimates muscle quantity and may itself be influenced by hydration status and fluid distribution in this population. The limited statistical power of these cross-sectional comparisons and the multiple comparisons performed further caution against overinterpretation. These findings indicate that functional screening tools complement, but cannot replace, objective muscle-mass measurement, and support imaging-based assessment where BIA reliability may be limited. In contrast, the proportion of patients classified as high-risk by MUST decreased significantly over the same interval (Table 4), even though muscle mass, handgrip strength, and albumin declined and CRP rose. This paradox likely reflects the structure of MUST, which is weighted toward acute unintentional weight loss and the acute-disease effect—components that are generally maximal around diagnosis and attenuate once patients stabilize on systemic treatment—compounded by the exclusion of the patients who died before month 6. MUST may therefore capture acute malnutrition risk rather than the slower, ongoing loss of muscle mass detected by SMI, and a declining MUST score during chemotherapy should not be interpreted as evidence of preserved muscle mass.

Muscle loss is a dynamic process rather than a static condition, and there is growing evidence that the longitudinal decline observed during treatment may carry prognostic value independent of muscle mass. In patients with metastatic colorectal cancer, SMI loss during palliative systemic treatment has been reported to be associated with early disease progression and shortened survival, whereas body mass index failed to reflect this muscle loss and to identify patients at risk [25]. The present study contributes to this framework by characterizing the dynamic decline in muscle mass in a gastrointestinal cancer cohort receiving chemotherapy and encompassing multiple tumor types, and by linking a circulating collagen turnover marker that accompanies this decline to the process; this approach raises the possibility of a complementary monitoring tool that is less dependent on imaging. Nevertheless, the limits of this inference must be stated clearly: survival and disease progression were not addressed as formal endpoints in the longitudinal analysis. Therefore, these findings demonstrate an association between a biomarker and the muscle mass trajectory but do not show a direct association of this marker with survival. Larger, prospective studies that evaluate survival and disease progression as predefined endpoints are needed to determine the prognostic value of PIIINP.

The main strengths of the present study are its prospective and longitudinal design, in contrast to the predominantly cross-sectional existing literature; the enrollment of consecutive patients, which limits selection bias; the reporting of muscle mass and biomarker changes in both absolute and proportional terms; and the application of false discovery rate correction for multiple comparisons. Furthermore, the cohort reflects a real-world gastrointestinal cancer population encompassing multiple tumor types.

From a clinical perspective, identifying progressive muscle loss during chemotherapy may have practical value beyond biochemical monitoring alone. Early recognition of patients with accelerating muscle loss could prompt timely, individualized interventions—such as targeted nutritional support and resistance-based physical activity or rehabilitation—rather than reliance on a single laboratory measurement. Whether a circulating marker such as PIIINP can usefully complement direct muscle assessment to identify these patients earlier warrants prospective evaluation. Accordingly, the proposal that PIIINP could serve as a complementary marker for monitoring muscle loss during chemotherapy should be regarded as hypothesis-generating and requires validation in prospective studies with predefined endpoints before any clinical application.

Several patient-level factors may influence both muscle mass and circulating PIIINP and were not fully controlled in this study. Comorbidities frequent in this population, such as diabetes and cardiovascular disease, are themselves associated with muscle loss [41], whereas fibrotic processes in other organs may raise circulating PIIINP independently of skeletal muscle. Medications may act in both directions: corticosteroids administered as chemotherapy premedication promote muscle catabolism [42], and cytotoxic regimens differ in their myotoxicity [43], yet cumulative corticosteroid exposure and regimen-specific effects were not systematically recorded; likewise, nutritional support, dietary counseling, and physical activity during chemotherapy were not recorded, and no standardized nutritional or exercise intervention was applied, so their influence on muscle loss and collagen turnover could not be assessed. These unmeasured factors—together with renal function, which is considered separately below—represent potential sources of residual confounding in the observed association between PIIINP and skeletal muscle loss.

This study has several limitations. First, the single-center design, the modest sample size, and the absence of an a priori power analysis limit generalizability; some non-significant findings—for example, the weak agreement of the screening tools with BIA-defined low muscle mass—may reflect limited statistical power rather than a true absence of association. Second, the 48 patients (28%) who died before month 6 could not be included in the longitudinal analyses, which are therefore susceptible to survivor bias; those who died were older and had a worse inflammatory, nutritional, and functional-screening profile, whereas baseline SMI and PIIINP were comparable (Tables S1 and S2), so their exclusion may have led to underestimation of muscle loss without operating through baseline muscle mass or PIIINP. Third, the month-6 albumin and CRP levels in the primary model were measured concurrently with the outcome; although a secondary model using baseline values (Table 7, Model 2) was consistent, reverse causation cannot be fully excluded. Fourth, the cohort was heterogeneous with respect to prior systemic treatment (first-line and subsequent-line patients), producing a non-uniform time-zero that may influence baseline muscle mass, collagen turnover, and the subsequent trajectory; cumulative corticosteroid exposure, chemotherapy toxicity, radiological tumor response, and disease progression—including whether patients remained on the index regimen—were not systematically recorded and could not be incorporated, although all surviving patients remained on systemic chemotherapy throughout, and although the expanded model adjusted for disease stage, tumor site, and treatment intent, residual confounding from line of therapy and these time-varying factors cannot be excluded. Fifth, BIA-derived muscle-mass estimates—and, to a lesser extent, anthropometric measures such as BMI—may be affected by hydration status and fluid distribution, which are common in gastrointestinal cancer (ascites, peripheral edema, hypoalbuminemia-related shifts); although patients with cirrhosis were excluded and standardized measurement conditions were applied, residual effects cannot be excluded, and because the Janssen equation was derived using tetrapolar hand-to-foot bioimpedance whereas the SC-330 uses a foot-to-foot configuration, absolute SMI values may be biased, although the reliance of the primary analyses on within-patient change measured with the same device and equation is expected to limit this influence. Sixth, defining the primary endpoint as percentage change carries a risk of sensitivity to extreme values and regression to the mean, particularly at low baseline values; consistent with this, baseline and month-6 PIIINP correlated with opposite signs with the percentage change in SMI (Table 6), most likely reflecting mathematical coupling through the shared denominator rather than independent biological effects, and reporting absolute changes supports interpretation but does not fully eliminate this limitation. Seventh, PIIINP is not specific to skeletal muscle and is also an established marker of hepatic fibrosis; in a cohort in which 64% had stage IV disease—frequently with hepatic metastases—and 114 of 122 patients (93%) received potentially hepatotoxic oxaliplatin- or irinotecan-based regimens, a hepatic contribution was a legitimate concern. To address this, liver function tests were measured at both time points and baseline liver-metastasis status was recorded, and a series of sensitivity analyses was performed (Section 3.7; Table 8): the inverse PIIINP–SMI association persisted in patients without liver metastasis (where it was, if anything, stronger), the change in PIIINP was not correlated with the change in any liver function test, and PIIINP remained independently associated with muscle loss after adjustment, in turn, for each baseline liver function test. These analyses argue against a predominantly hepatic origin; nevertheless, because PIIINP lacks tissue specificity and dedicated fibrogenesis markers (e.g., the enhanced liver fibrosis panel) and liver histology were not assessed, a minor hepatic contribution cannot be entirely excluded. Finally, circulating PIIINP may be influenced by renal dysfunction; although patients with end-stage chronic kidney disease were excluded, six with non-end-stage disease were included, and excluding them in a sensitivity analysis did not materially alter the primary association, though a residual influence of renal function cannot be excluded.

## 5. Conclusions

This prospective longitudinal study showed that the skeletal muscle index (SMI) decreased significantly over a six-month follow-up period in patients with gastrointestinal cancer receiving chemotherapy, and that this decrease was accompanied by an increase in levels of the circulating collagen turnover marker PIIINP. The percentage change in PIIINP was strongly and inversely associated with the percentage change in SMI, and this association remained significant across both parsimonious and expanded multivariable models. Given the observational design and the lack of tissue specificity of circulating PIIINP, these findings should not be interpreted as evidence of a causal or muscle-specific pathophysiological mechanism. Rather, PIIINP should be regarded as a candidate biomarker whose longitudinal association with muscle loss requires independent validation. Such validation should use standardized measures of muscle mass, including imaging-based approaches such as the CT-derived skeletal muscle index, together with clinically defined sarcopenia, in larger, multicenter prospective cohorts. Mechanistic studies are also needed to determine the biological basis of the observed association.

## Figures and Tables

**Figure 1:**
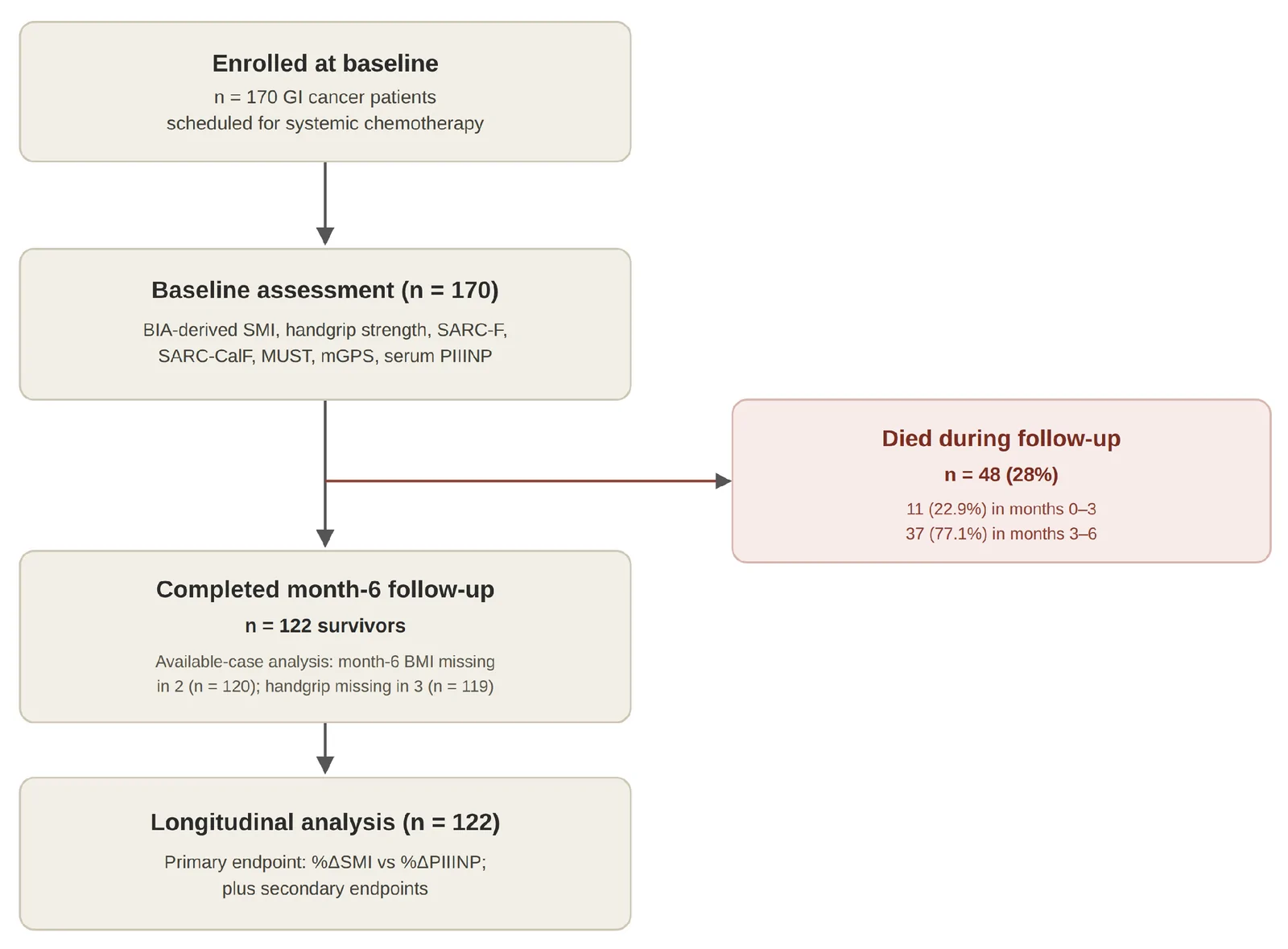
Study flow diagram. Of 170 patients with gastrointestinal cancer enrolled and assessed at baseline, 48 (28%) died during the six-month follow-up and 122 survivors completed the month-6 assessment and were included in the longitudinal analyses. Month-6 values were missing for body mass index in 2 patients and handgrip strength in 3 patients; the corresponding paired analyses used an available-case approach. SMI, skeletal muscle index; PIIINP, procollagen type III *N*-terminal peptide; mGPS, modified Glasgow Prognostic Score; BIA, bioelectrical impedance analysis; BMI, body mass index; GI, gastrointestinal; MUST, Malnutrition Universal Screening Tool; SARC-CalF, SARC-F plus calf circumference; SARC-F, strength, assistance walking, rising from a chair, climbing stairs, and falls.

**Figure 2:**
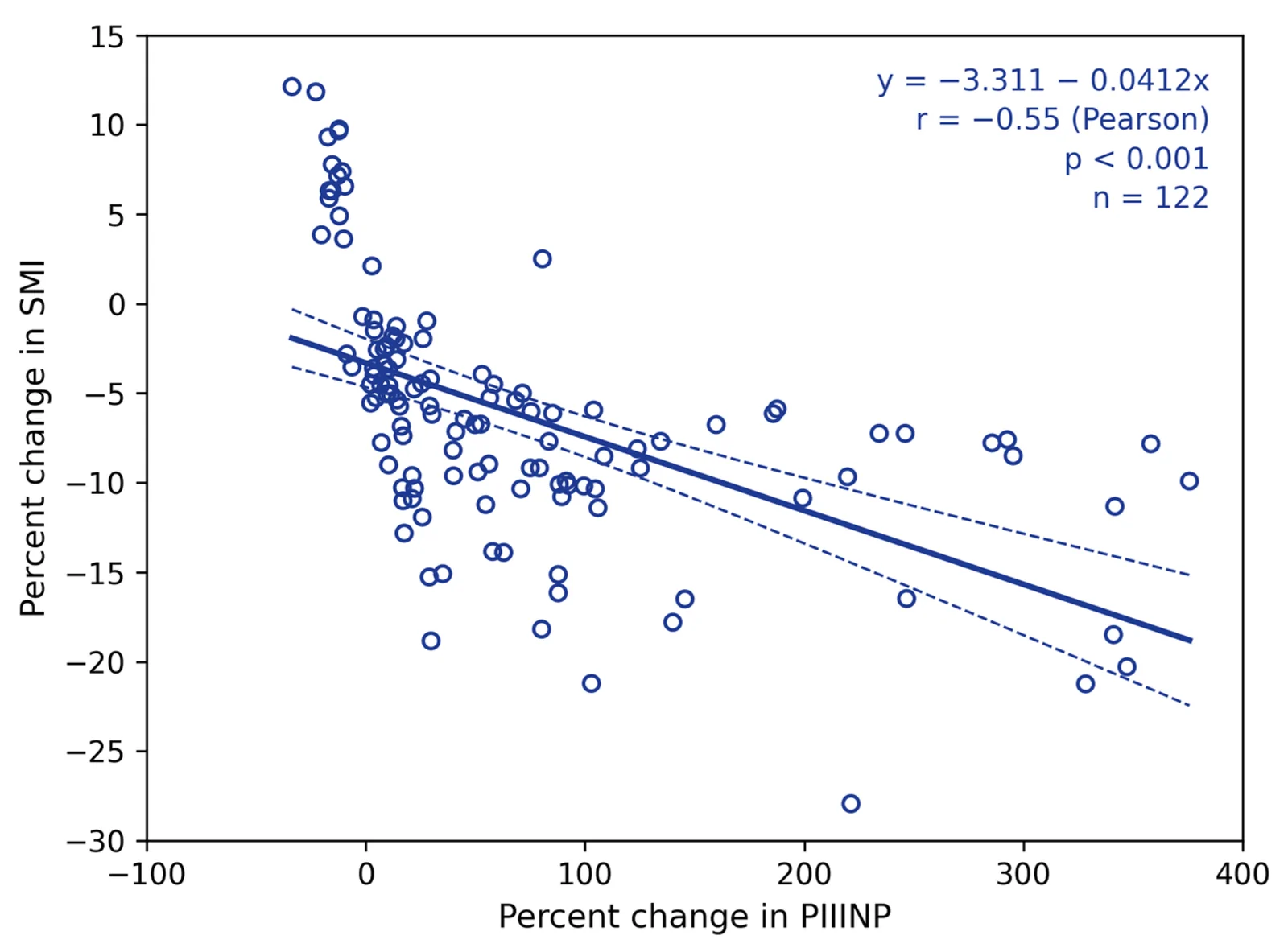
Scatter plot of the percentage change in serum PIIINP against the percentage change in the skeletal muscle index (SMI) between baseline and month 6 (*n* = 122). Each point represents an individual patient; the solid line shows the ordinary least-squares regression fit (y = −3.311 − 0.0412x) with its 95% confidence interval (dashed lines). The percentage changes were inversely correlated (Spearman ρ = −0.733, *p* < 0.001); the Pearson coefficient was more modest (r = −0.55, *p* < 0.001), consistent with the right-skewed distribution of the percentage change in PIIINP. The association is distributed across the cohort rather than driven by a few extreme observations. PIIINP, procollagen type III *N*-terminal peptide; SMI, skeletal muscle index.

**Figure 3:**
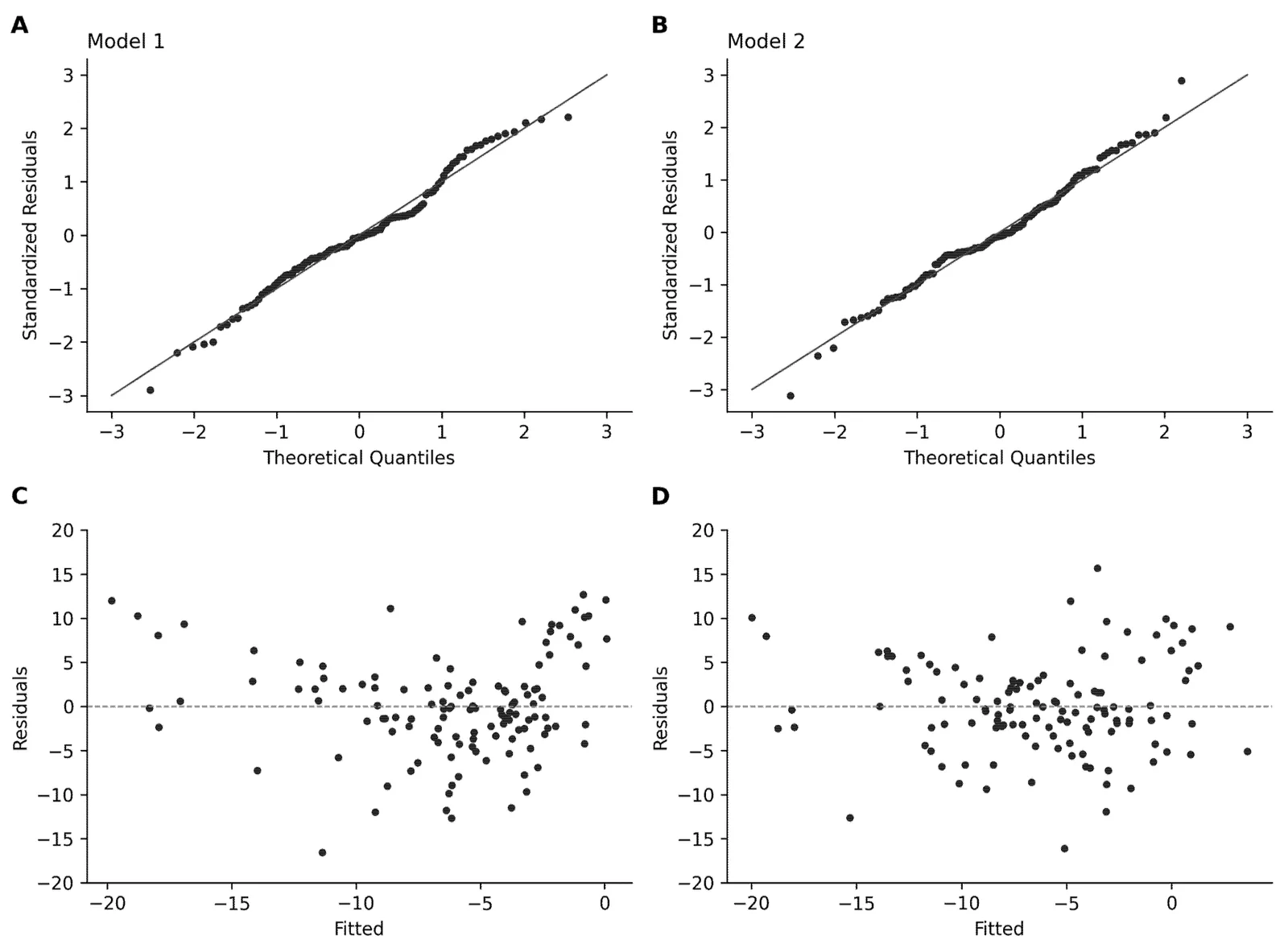
Residual diagnostic plots for the multiple linear regression models predicting the percentage change in the skeletal muscle index (Table 7). (**A**,**B**) Normal quantile–quantile plots of the standardized residuals for Model 1 (**A**) and Model 2 (**B**); the residuals approximate the reference line, consistent with normality. (**C**,**D**) Residuals versus fitted values for Model 1 (**C**) and Model 2 (**D**), showing no systematic pattern, consistent with linearity and homoscedasticity. Model assumptions were formally confirmed (Shapiro–Wilk, Breusch–Pagan, and Durbin–Watson tests), and all variance inflation factors were below 5.

**Table 1:** Demographic and clinical characteristics of patients at baseline (*n* = 122).

Parameter	*n* (%)/Mean ± SD
**Sex, *n* (%)**	
Male	82 (67)
Female	40 (33)
Age (years), mean ± SD	60.3 ± 10.4
**Age group, *n* (%)**	
≥65 years	42 (34)
<65 years	80 (66)
**Any chronic comorbidity, *n* (%)**	44 (36)
Hypertension	9 (7)
Diabetes mellitus	18 (15)
Coronary artery disease	12 (10)
Chronic kidney disease	6 (5)
COPD	3 (3)
BMI (kg/m^2^), mean ± SD	24.8 ± 4.8
**BMI category, *n* (%)**	
Underweight (<18.5)	8 (7)
Normal (18.5–24.9)	53 (43)
Overweight (25.0–29.9)	44 (36)
Obese (≥30.0)	17 (14)

Data are presented as *n* (%) or mean ± standard deviation (SD). Percentages of individual comorbidities may sum to more than the “any chronic comorbidity” total because some patients had more than one comorbidity. BMI categories were defined according to World Health Organization criteria. BMI, body mass index; COPD, chronic obstructive pulmonary disease; SD, standard deviation.

**Table 2:** Oncological characteristics of patients (*n* = 122).

Parameter	*n* (%)
**Primary tumor site, *n* (%)**	
Colon	59 (48)
Rectum	26 (21)
Gastric	26 (21)
Other	11 (9)
**Disease stage, *n* (%)**	
Stage II	11 (9)
Stage III	33 (27)
Stage IV	78 (64)
**Liver metastasis, *n* (%)**	
Present	36 (30)
Absent	86 (70)
**Treatment intent, *n* (%)**	
Adjuvant	23 (19)
Neoadjuvant	20 (16)
Palliative	79 (65)
**Chemotherapy regimen, *n* (%)**	
FOLFOX	68 (56)
FOLFIRI	18 (15)
XELOX	18 (15)
FLOT	9 (7)
FOLFIRINOX	1 (1)
Other regimens	8 (7)

Data are presented as *n* (%). Disease stage was determined according to the AJCC Cancer Staging Manual, 8th edition. “Other” tumor sites included esophageal, pancreatic, biliary tract, and small-bowel tumors. “Other regimens” comprised gemcitabine + capecitabine (*n* = 3), gemcitabine + cisplatin (*n* = 2), gemcitabine (*n* = 2), and paclitaxel + carboplatin (*n* = 1). AJCC, American Joint Committee on Cancer. FLOT, fluorouracil, folinic acid, oxaliplatin, and docetaxel; FOLFIRI, folinic acid, fluorouracil, and irinotecan; FOLFIRINOX, folinic acid, fluorouracil, irinotecan, and oxaliplatin; FOLFOX, folinic acid, fluorouracil, and oxaliplatin; XELOX, capecitabine and oxaliplatin. Liver metastasis denotes radiologically confirmed hepatic metastatic disease at baseline and represents a subset of stage IV disease, as the remaining patients with stage IV disease had metastases at extrahepatic sites.

**Table 3:** Changes in anthropometric, laboratory, and inflammatory parameters between baseline and month 6 (*n* = 122).

Parameter	Baseline	Month 6	Δ (Change)	*p*
BMI (kg/m^2^), mean ± SD ^f^	24.81 ± 4.80	23.68 ± 5.04	−1.13 ± 1.78	**<0.001** ^b^
Creatinine (mg/dL) ^g^, median (IQR)	0.76 (0.64–0.88)	0.75 (0.70–0.86)	0.015 (−0.005 to 0.040) ^a^	0.179 ^a^
C-reactive protein (mg/L) ^g^, median (IQR)	5.0 (2.0–12.0)	6.5 (4.0–12.0)	1.75 (0.00 to 2.50) ^a^	**0.034** ^a^
Albumin (g/L) ^g^, median (IQR)	41 (38–43)	38 (36–40)	−2.5 (−3.0 to −2.0) ^a^	**<0.001** ^a^
**mGPS score, *n* (%)**			—	**0.013** ^c^
Score 0	85 (70)	81 (66)		
Score 1	32 (26)	24 (20)		
Score 2	5 (4)	17 (14)		

Data are presented as mean ± SD, median (IQR), or *n* (%), as appropriate. Bold *p* values indicate statistical significance (*p* < 0.05). Δ values for BMI are mean differences (paired-samples *t*-test); Δ values for creatinine, CRP, and albumin are Hodges–Lehmann median differences with 95% CI, paired with the Wilcoxon signed-rank test. ^a^ Wilcoxon signed-rank test; ^b^ paired-samples *t*-test; ^c^ McNemar–Bowker test; ^f^ BMI at month 6 was missing for two patients due to incomplete anthropometric assessment; the paired comparison was based on *n* = 120; ^g^ institutional reference ranges: creatinine, 0.7–1.2 mg/dL (male)/0.5–1.0 mg/dL (female); CRP, 0–5 mg/L; albumin, 35–52 g/L. BMI, body mass index; CI, confidence interval; CRP, *C*-reactive protein; IQR, interquartile range; mGPS, modified Glasgow Prognostic Score; SD, standard deviation.

**Table 4:** Changes in sarcopenia-related parameters and nutritional screening scores between baseline and month 6 (*n* = 122).

Parameter	Baseline	Month 6	Δ (Change)	% Change	*p*
SMI (kg/m^2^), mean ± SD	10.14 ± 1.75	9.50 ± 1.76	−0.64 ± 0.80	−6.2 ± 7.1	**<0.001** ^b^
PIIINP (pg/mL), mean ± SD	1521.5 ± 900.9	2071.6 ± 888.6	550.1 ± 551.8	70.6 ± 95.5	**<0.001** ^b^
Handgrip strength (kg) ^f^, median (IQR)	32 (22–36)	26 (20–34)	−2.5 (−3.0 to −2.0) ^a^	−9.09 (−16.67 to 0.00)	**<0.001** ^a^
SARC-F score, median (IQR)	0 (0–2)	1 (0–3)	0.5 (0.5 to 1.0) ^a^	— ^c^	**<0.001** ^a^
**SARC-F risk category, *n* (%)**					0.453 ^d^
High risk (≥4)	19 (16)	21 (17)			
Low risk (<4)	103 (84)	100 (83)			
SARC-CalF score, median (IQR)	0 (0–2)	1 (0–4)	0.5 (0.5 to 1.0) ^a^	— ^c^	**<0.001** ^a^
**SARC-CalF risk category, *n* (%)**					**0.031** ^d^
High risk (≥11)	17 (14)	23 (19)			
Low risk (<11)	105 (86)	98 (81)			
**MUST risk category, *n* (%)**					**0.003** ^e^
Low risk (0)	44 (36)	54 (45)			
Medium risk (1)	36 (30)	46 (38)			
High risk (≥2)	42 (34)	21 (17)			

Data are presented as mean ± SD, median (IQR), or *n* (%), as appropriate. Bold *p* values indicate statistical significance (*p* < 0.05). Δ values for SMI and PIIINP are mean differences (paired-samples *t*-test); Δ values for handgrip strength and SARC-F/SARC-CalF scores are Hodges–Lehmann median differences with 95% CI, paired with the Wilcoxon signed-rank test. ^a^ Wilcoxon signed-rank test; ^b^ paired-samples *t*-test; ^c^ percentage change was not calculated for SARC-F and SARC-CalF because the baseline score was 0 in a substantial proportion of patients, rendering percentage change mathematically undefined; ^d^ McNemar test; ^e^ McNemar–Bowker test; ^f^ handgrip strength was missing for three patients at one time point; the paired comparison was based on *n* = 119. IQR, interquartile range; MUST, Malnutrition Universal Screening Tool; PIIINP, procollagen type III *N*-terminal peptide; SARC-CalF, SARC-F plus calf circumference; SARC-F, strength, assistance walking, rising from a chair, climbing stairs, and falls; SD, standard deviation; SMI, skeletal muscle index.

**Table 5:** Association between malnutrition and sarcopenia screening tools and BIA-defined low muscle mass at baseline and month 6 (*n* = 122).

Parameter	Baseline		*p*	Month 6		*p*
	Low Muscle Mass, *n* (%)	Normal, *n* (%)		Low Muscle Mass, *n* (%)	Normal, *n* (%)	
**MUST score**			0.798 ^a^			0.465 ^a^
Low risk (0)	14 (40)	30 (35)		25 (40)	29 (50)	
Medium risk (1)	9 (26)	27 (31)		27 (43)	19 (33)	
High risk (≥2)	12 (34)	30 (35)		11 (17)	10 (17)	
**SARC-F risk category**			0.103 ^b^			0.491 ^b^
Low risk (<4)	33 (94)	70 (81)		54 (86)	46 (79)	
High risk (≥4)	2 (6)	17 (20)		9 (14)	12 (21)	
**SARC-CalF risk category**			0.827 ^b^			0.479 ^b^
Low risk (<11)	31 (89)	74 (85)		49 (78)	49 (84)	
High risk (≥11)	4 (11)	13 (15)		14 (22)	9 (16)	

Values are *n* (%); percentages are column percentages (i.e., within each muscle-mass category). Low muscle mass was defined by BIA-derived SMI (<10.76 kg/m^2^ in men; <6.76 kg/m^2^ in women). Month-6 analyses were based on 121 patients, because month-6 screening data were missing for one patient (available-case analysis). ^a^ Pearson chi-square test; ^b^ chi-square test with Yates continuity correction. BIA, bioelectrical impedance analysis; MUST, Malnutrition Universal Screening Tool; SARC-CalF, SARC-F plus calf circumference; SARC-F, strength, assistance walking, rising from a chair, climbing stairs, and falls; SMI, skeletal muscle index.

**Table 6:** Factors associated with the percent change in SMI (Spearman correlation analysis).

Parameter	Time Point	rho	*p*-Value
Percent change in PIIINP	Δ (month 6 − baseline)	−0.733	**<0.001**
Albumin	Month 6	0.479	**<0.001**
C-reactive protein	Month 6	−0.391	**<0.001**
SARC-CalF score	Month 6	−0.253	**0.005**
PIIINP	Baseline	0.236	**0.009**
Handgrip strength	Month 6	0.234	**0.009**
SARC-F score	Month 6	−0.220	**0.015**
PIIINP	Month 6	−0.218	**0.016**
BMI	Month 6	0.171	0.059
BIA resistance (R)	Baseline	0.138	0.129
Albumin	Baseline	−0.111	0.222
Creatinine	Month 6	0.098	0.283
SARC-F score	Baseline	0.053	0.563
Creatinine	Baseline	−0.031	0.733
SARC-CalF score	Baseline	0.028	0.756
BMI	Baseline	−0.020	0.827
Age	Baseline	−0.014	0.878
C-reactive protein	Baseline	0.008	0.928
Handgrip strength	Baseline	−0.005	0.961

Nineteen candidate variables were tested. All associations significant at *p* < 0.05 (shown in bold) remained significant after Benjamini–Hochberg false discovery rate correction. The Spearman coefficient (rho) was interpreted as 0.00–0.19, very weak; 0.20–0.39, weak; 0.40–0.59, moderate; 0.60–0.79, strong; and 0.80–1.00, very strong; negative values indicate an inverse association. Percent change was calculated as [(month-6 value − baseline value)/baseline value] × 100. For the PIIINP–SMI association specifically, the Pearson correlation coefficient was weaker (r = −0.55, *p* < 0.001; see Figure 2), reflecting the right-skewed distribution of the percentage change in PIIINP. BIA, bioelectrical impedance analysis; BMI, body mass index; PIIINP, procollagen type III *N*-terminal peptide; SARC-CalF, SARC-F plus calf circumference; SARC-F, strength, assistance walking, rising from a chair, climbing stairs, and falls; SMI, skeletal muscle index. The Spearman correlation between the absolute changes in SMI and PIIINP (month 6 − baseline) was ρ = −0.722 (*p* < 0.001), closely matching the percentage-change coefficient and indicating that the association is not an artifact of the percentage transformation.

**Table 7:** Multiple linear regression analysis for factors associated with the percent change in SMI.

	Model 1				Model 2			
Predictor	B (95% CI)	β	*p*	VIF	B (95% CI)	β	*p*	VIF
Constant	−26.26 (−39.54 to −12.97)	—	**<0.001**	—	15.38 (−7.65 to 38.40)	—	0.188	—
% Change in PIIINP	−0.03 (−0.05 to −0.02)	−0.47	**<0.001**	1.16	−0.043 (−0.058 to −0.028)	−0.57	**<0.001**	1.40
Albumin (Month 6/Baseline ^d^)	0.58 (0.25 to 0.91)	0.31	**0.001**	1.45	−0.202 (−0.556 to 0.151)	−0.10	0.258	1.28
CRP (Month 6/Baseline ^d^)	0.05 (−0.06 to 0.16)	0.08	0.349	1.42	0.006 (−0.073 to 0.084)	0.01	0.886	1.18
Age	—	—	—	—	−0.085 (−0.195 to 0.024)	−0.12	0.126	1.11
Sex (Female vs. Male)	—	—	—	—	−1.757 (−4.704 to 1.190)	−0.24	0.240	1.35
Disease Stage (III vs. II)	—	—	—	—	0.510 (−3.725 to 4.746)	0.07	0.812	2.48
Disease Stage (IV vs. II)	—	—	—	—	6.448 (−6.638 to 19.534)	0.90	0.331	2.48
SMI, baseline	—	—	—	—	−1.152 (−1.972 to −0.332)	−0.28	**0.006**	1.40
PIIINP, baseline	—	—	—	—	−0.001 (−0.002 to 0.001)	−0.08	0.447	1.41
Treatment Intent (Adjuvant vs. Palliative)	—	—	—	—	10.931 (−1.438 to 23.299)	1.52	0.083	2.63
Treatment Intent (Neoadjuvant vs. Palliative)	—	—	—	—	7.128 (−5.877 to 20.134)	0.99	0.280	2.63
Tumor Site (Rectum vs. Colon)	—	—	—	—	2.150 (−0.905 to 5.205)	0.30	0.166	1.12
Tumor Site (Gastric vs. Colon)	—	—	—	—	0.581 (−2.413 to 3.575)	0.08	0.701	1.12
Tumor Site (Other vs. Colon)	—	—	—	—	−1.551 (−5.680 to 2.578)	−0.22	0.458	1.12

B, unstandardized regression coefficient; CI, confidence interval; VIF, variance inflation factor; β, standardized regression coefficient. Bold *p* values indicate statistical significance (*p* < 0.05). Model 1 (parsimonious) values correspond to the originally reported model. Model 2 (expanded) additionally adjusted for age, sex, disease stage, baseline SMI, baseline PIIINP, treatment intent, and primary tumor site; ^d^ baseline albumin and CRP were used in Model 2 instead of month-6 values to avoid reverse-causation bias. Each model’s VIF values were computed within its own model specification; because Model 1 and Model 2 include different covariate sets, the VIF for a given predictor (e.g., PIIINP) differs between models and is reported separately for each. Because Model 2 substitutes baseline for month-6 values for two predictors rather than simply adding covariates, Models 1 and 2 are not nested and are therefore not compared via a formal F-change test; each model’s fit is reported independently. The omnibus test for treatment intent was significant (*p* = 0.046) despite no individual pairwise comparison reaching significance. Model assumptions were checked with residual analyses (Model 1: Shapiro–Wilk *p* = 0.106, Breusch–Pagan *p* = 0.577, Durbin–Watson *p* = 0.856; Model 2: Shapiro–Wilk *p* = 0.661, Breusch–Pagan *p* = 0.081, Durbin–Watson *p* = 0.900), all satisfied; all VIF values were below 5 in both models. A sensitivity analysis using natural log-transformed values (Δln[PIIINP], Δln[SMI]) was consistent with the primary analysis (β = −0.59, *p* < 0.001, R^2^ = 0.46; see Table S3). PIIINP, procollagen type III *N*-terminal peptide; SMI, skeletal muscle index.

**Table 8:** Hepatic sensitivity analyses evaluating the potential contribution of liver function to the association between the six-month percentage changes in PIIINP and SMI (*n* = 122).

Analysis	*n*	Value	*p*
**A. Correlation of %ΔPIIINP with %ΔSMI, stratified by baseline liver metastasis (ρ)**			
Overall	122	−0.733	<0.001
Without liver metastasis	86	−0.812	<0.001
With liver metastasis	36	−0.485	0.003
**B. Correlation of %ΔPIIINP with six-month change in liver function test (ρ)**			
ΔALT	122	−0.070	0.440
ΔAST	122	−0.008	0.928
ΔGGT	122	−0.125	0.169
ΔALP	122	−0.137	0.131
ΔTotal bilirubin	122	−0.073	0.427
**C. Six-month change in liver function test, Wilcoxon signed-rank (median: baseline → month 6)**			
ALT (U/L)	122	24.0 → 24.0	0.049
AST (U/L)	122	23.0 → 25.0	0.014
GGT (U/L)	122	46.0 → 46.0	0.832
ALP (U/L)	122	81.0 → 83.0	0.436
Total bilirubin (mg/dL)	122	0.4 → 0.5	0.321
**D. Model 2 re-fitted with each baseline liver function test added (β PIIINP/β test)**			
+baseline ALT	122	−0.57/−0.08	<0.001/0.310
+baseline AST	122	−0.58/−0.14	<0.001/0.073
+baseline GGT	122	−0.57/−0.03	<0.001/0.667
+baseline ALP	122	−0.57/−0.03	<0.001/0.725
+baseline total bilirubin	122	−0.57/+0.05	<0.001/0.537

PIIINP, procollagen type III *N*-terminal peptide; SMI, skeletal muscle index; ρ, Spearman correlation coefficient; β, standardized regression coefficient; ALT, alanine aminotransferase; AST, aspartate aminotransferase; GGT, gamma-glutamyl transferase; ALP, alkaline phosphatase. All panels are based on the 122 patients who completed both the baseline and month-6 assessments. In Panel B, Δ denotes the six-month change (month-6 minus baseline value). In Panel C, values are the baseline and month-6 medians among completers; neither the ALT nor the AST change remained significant after Bonferroni correction for the five comparisons (α = 0.010), and all medians remained within the reference range. In Panel D, each baseline liver function test was entered separately into the expanded model (Model 2) rather than simultaneously, to avoid increasing the number of model parameters and collinearity between functionally related enzymes (ALT–AST and ALP–GGT); β PIIINP and β test denote the standardized coefficients for the percentage change in PIIINP and for the added liver function test, respectively.

## Data Availability

The data presented in this study are available on request from the corresponding author due to privacy and ethical restrictions.

## Supplementary Materials

The following supporting information can be downloaded at https://www.mdpi.com/article/10.3390/nu18193149/s1, Table S1: Comparison of Demographic, Functional, and Biochemical Baseline Characteristics Between Survivors and Non-survivors (*n* = 170). Table S2: Comparison of Oncological and Treatment-Related Baseline Characteristics Between Survivors and Non-survivors (*n* = 170). Table S3: Sensitivity Analysis: Multiple Linear Regression Using Natural Log-Transformed PIIINP and SMI Values. Table S4: Stability of the Standardized PIIINP Coefficient Across Regression Specifications.
