# Supplementary material for: Longitudinal Association Between the Change in Serum Procollagen Type III N-Terminal Peptide (PIIINP) and Skeletal Muscle Loss in Patients with Gastrointestinal Cancer Receiving Chemotherapy: A Prospective Cohort Study
Source: Nutrients. 2026 Sep 24;18(19):3149. doi: 10.3390/nu18193149 (PMC13667261; doi:10.3390/nu18193149)
Supplement: Supplementary file 1 [file nutrients-18-03149-s001.zip › nutrients-4520177-supplementary.pdf]

Supplementary Materials

Table S1. Comparison of Demographic, Functional, and Biochemical Baseline Characteristics Between Survivors and Non-survivors (*n* = 170).

Parameter	Total (*n* = 170)	Alive (*n* = 122)	Deceased (*n* = 48)	*p*
Male, *n* (%)	112 (66)	82 (67)	30 (63)	0.686 [d]
Age (years), median (IQR)	62.5 (56–69)	60.5 (55–68)	66.5 (60–72.5)	**0.002** [a]
Age ≥65 years, *n* (%)	70 (41)	42 (34)	28 (58)	**0.007 d**
Any chronic comorbidity, *n* (%)	66 (39)	42 (34)	24 (50)	0.089 [d]
Hypertension	10 (6)	5 (4)	5 (10)	0.147 [e]
Diabetes mellitus	29 (17)	18 (15)	11 (23)	0.295 [d]
Coronary artery disease	20 (12)	12 (10)	8 (17)	0.327 [d]
Chronic kidney disease	8 (5)	6 (5)	2 (4)	1.000 [e]
COPD	5 (3)	3 (2)	2 (4)	0.622 [e]
BMI (kg/m^2), median (IQR)	24 (21–27)	24.85 (21–27.4)	23.35 (21.05–25)	0.113 [a]
**BMI category, *n* (%)**				**0.037 c**
Underweight (<18.5)	11 (6)	8 (7)	3 (6)	
Normal (18.5–24.9)	85 (50)	53 (43)	32 (67)	
Overweight (25.0–29.9)	55 (32)	44 (36)	11 (23)	
Obese (≥30.0)	19 (11)	17 (14)	2 (4)	
Creatinine (mg/dL), median (IQR)	0.76 (0.60–0.90)	0.76 (0.64–0.88)	0.80 (0.60–0.90)	0.755 [a]
C-reactive protein (mg/L), median (IQR)	6 (2–16)	5 (2–12)	14 (6–33.5)	**<0.001** [a]
Albumin (g/L), median (IQR)	40 (38–42)	40.5 (38–43)	38.5 (36–40)	**<0.001** [a]
**mGPS score, *n* (%)**				**<0.001** [c]
Score 0	104 (61)	85 (70)	19 (40)	
Score 1	53 (31)	32 (26)	21 (44)	
Score 2	13 (8)	5 (4)	8 (17)	
PIIINP (pg/mL), median (IQR)	1498.6 (881.4–2195.3)	1504.6 (674.8–2195.3)	1362.7 (963.5–2201.0)	0.428 [a]

Parameter	Total (*n* = 170)	Alive (*n* = 122)	Deceased (*n* = 48)	*p*
BIA resistance (Ω), median (IQR)	439 (394–495)	435 (394–484)	445.5 (393.5–507.5)	0.587 [a]
SMI (kg/m^2), mean ± SD	10.14 ± 1.77	10.14 ± 1.75	10.13 ± 1.84	0.980 [b]
BIA-defined low muscle mass, *n* (%)	50 (29)	35 (29)	15 (31)	0.886 [d]
Handgrip strength (kg), median (IQR)	30 (22–36)	32 (22–36)	27 (19–34)	0.109 [a]
MUST score, median (IQR)	1 (0–2)	1 (0–2)	1 (1–2)	0.118 [a]
SARC-F score, median (IQR)	0 (0–3)	0 (0–2)	2 (0–4)	**<0.001** [a]
SARC-F high risk (≥4), *n* (%)	39 (23)	19 (16)	20 (42)	**<0.001** [d]
SARC-CalF score, median (IQR)	0 (0–4)	0 (0–2)	2 (0–5)	**<0.001** [a]
SARC-CalF high risk (≥11), *n* (%)	26 (15)	17 (14)	9 (19)	0.583 [d]

Continuous variables are presented as median (IQR) or mean ± SD, as appropriate; categorical variables as n (%). Low muscle mass was defined by BIA-derived SMI (<10.76 kg/m^2 in men; <6.76 kg/m^2 in women). BMI categories were defined according to World Health Organization criteria. a Mann-Whitney U test; b independent-samples t-test; c Pearson chi-square test; d chi-square test with Yates' continuity correction; e Fisher's exact test. BIA, bioelectrical impedance analysis; BMI, body mass index; COPD, chronic obstructive pulmonary disease; IQR, interquartile range; mGPS, modified Glasgow Prognostic Score; MUST, Malnutrition Universal Screening Tool; PIIINP, procollagen type III N-terminal peptide; SARC-CalF, SARC-F plus calf circumference; SARC-F, Strength, Assistance walking, Rising from a chair, Climbing stairs, and Falls; SD, standard deviation; SMI, skeletal muscle index.

Table S2. Comparison of Oncological and Treatment-Related Baseline Characteristics Between Survivors and Non-survivors (*n* = 170).

Parameter	Total (*n* = 170)	Alive (*n* = 122)	Deceased (*n* = 48)	*p*
**Primary tumor site, *n* (%)**				0.413 [c]
Colon	78 (46)	59 (48)	19 (40)	
Rectum	34 (20)	26 (21)	8 (17)	
Gastric	40 (24)	26 (21)	14 (29)	
Other	18 (11)	11 (9)	7 (15)	
**Disease stage, *n* (%)**				0.330 [c]
Stage II	13 (8)	11 (9)	2 (4)	
Stage III	43 (25)	33 (27)	10 (21)	
Stage IV	114 (67)	78 (64)	36 (75)	
**Treatment intent, *n* (%)**				0.298 [c]
Adjuvant	29 (17)	23 (19)	6 (13)	
Neoadjuvant	25 (15)	20 (16)	5 (10)	
Palliative	116 (68)	79 (65)	37 (77)	
**Chemotherapy regimen, *n* (%)**				0.094 [f]
FOLFOX	95 (56)	68 (56)	27 (56)	
XELOX	22 (13)	18 (15)	4 (8)	
FOLFIRI	23 (14)	18 (15)	5 (10)	
FLOT	11 (6)	9 (7)	2 (4)	
FOLFIRINOX	5 (3)	1 (1)	4 (8)	
Other regimens	14 (8)	8 (7)	6 (13)	

Data are presented as n (%). Disease stage was determined according to the AJCC Cancer Staging Manual, 8th edition. c Pearson chi-square test; f Fisher–Freeman–Halton exact test (used because more than 20% of cells had an expected count below 5). AJCC, American Joint Committee on Cancer.

Table S3. Sensitivity Analysis: Multiple Linear Regression Using Natural Log-Transformed PIIINP and SMI Values.

Predictor	B (95% CI)	β	t	*p*	VIF
Intercept	−0.216 (−0.351 to −0.080)	—	−3.15	**0.002**	—
Δ ln(PIIINP)	−0.098 (−0.122 to −0.073)	−0.59	−8.00	**<0.001**	1.20
CRP, month 6	0.001 (−0.000 to 0.002)	0.09	1.15	0.251	1.41
Albumin, month 6	0.005 (0.001 to 0.008)	0.23	2.84	**0.005**	1.49

Dependent variable: Δ ln(SMI), calculated as ln(month-6 SMI) − ln(baseline SMI). Δ ln(PIIINP), calculated as ln(month-6 PIIINP) − ln(baseline PIIINP), was substituted for percentage change in PIIINP to address the right-skewed distribution of the latter (see Table 7). B, unstandardized regression coefficient; CI, confidence interval; VIF, variance inflation factor; β, standardized regression coefficient. Model assumptions were checked with residual analyses (Shapiro-Wilk p=0.140; Breusch-Pagan p=0.203; Durbin-Watson p=0.778), all satisfied; all VIF values were below 5, indicating no relevant multicollinearity. Compared with the primary model (Table 7, Model 1), this log-transformed sensitivity analysis yielded a stronger association for PIIINP (β=−0.59 vs. −0.47) and a higher model fit (R^2=0.46 vs. 0.37), confirming that the PIIINP–SMI association is not an artifact of data skewness. CRP, C-reactive protein; PIIINP, procollagen type III N-terminal peptide; SMI, skeletal muscle index. Model fit: R=0.68, R^2=0.46, adjusted R^2=0.45, F(3,118)=33.61, p<0.001.

Table S4. Stability of the Standardized PIIINP Coefficient Across Regression Specifications.

Specification	Predictors, *n*	PIIINP standardized β
Model 1 (parsimonious)	3	−0.47
Model 2 (expanded)	14	−0.57
Ridge (penalized)	14	−0.46
LASSO (penalized)	14 (3 retained)	−0.44

β denotes the standardized regression coefficient for the percentage change in PIIINP. Model 1 and Model 2 correspond to the parsimonious and expanded ordinary least-squares models reported in Table 7. Ridge and LASSO are penalized regression models fitted to the Model 2 predictor set, with the shrinkage parameter selected by 10-fold cross-validation. In the LASSO model, PIIINP was retained as the predictor with the largest coefficient, together with baseline SMI and palliative treatment intent, while all other covariates were shrunk to zero. The stability of the PIIINP coefficient across all four specifications indicates that it is not an artifact of overfitting. LASSO, least absolute shrinkage and selection operator; PIIINP, procollagen type III N-terminal peptide; SMI, skeletal muscle index.
